# Prevalence of Amlodipine-induced gingival enlargement in a Tertiary Care Hospital: A Descriptive Cross-Sectional Study

**DOI:** 10.31729/jnma.8989

**Published:** 2025-05-31

**Authors:** Gitanjali Subedi, Arjun Hari Rijal, Simant Lamichhane, Osha Ghimire, Manoj Humagain

**Affiliations:** 1 Department of Periodontology and Oral Implantology, Kathmandu University School of Medical Sciences, Dhulikhel, Kavrepalanchowk, Nepal; 2 Department of Dentistry, Kathmandu University School of Medical Sciences, Dhulikhel, Kavrepalanchowk, Nepal

**Keywords:** *amlodipine*, *drug dosage*, *gingival enlargement*, *severity*

## Abstract

**Introduction:**

Administration of certain drugs such as anticonvulsants, immunosuppressants, and calcium channel blockers leads to well-known sequalae of gingival enlargement. Apart from esthetic consequences, gingival enlargement also hinders proper oral hygiene and may be painful for the patient. Determination of primary etiology and subsequent treatment for the same is pre-requisite in the management of gingival enlargement. Therefore, this study was intended to determine the prevalence of gingival enlargement in patients under amlodipine therapy in a tertiary care hospital.

**Methods:**

A descriptive cross-sectional study was carried out after ethical approval from Institutional Review Committee (Reference No: 242/23), in the Department of Internal Medicine and in the Department of Periodontology, Dhulikhel Hospital. The study period was from December, 2023 - May, 2024. Patients under amlodipine therapy for at least six months were assessed. Purposive sampling was done with a total sample size of 450. The prevalence of gingival enlargement along with dosage and duration of drug intake was calculated.

**Results:**

Out of 450 participants, drug induced gingival enlargement was seen in 129 (28.70%). Furthermore, among 129 patients, grade 1 gingival enlargement was more prevalent (n=93, 20.70%) while grade 3 gingival enlargement was only seen in 6 (0.90%) of the participants. The dosage and duration of drug intake by patient were also calculated.

**Conclusions:**

Gingival enlargement could be the potential side effect associated with amlodipine usage. It was found to be prevalent in patients under amlodipine therapy. The patients should be well-informed about the consequence, by the treating physician, prior to initiation of amlodipine therapy. Proper oral hygiene measures should be reinforced in such patients from the beginning.

## INTRODUCTION

Gingival enlargement,oneofthe most significantcharacteristics of gingival disease, has multifactorial etiology and may show up as a variety of clinical traits. Administration of certain drugs such as anticonvulsants, immunosuppressants, and calcium channel blockers have been shown to be associated with a well-known consequence of gingival enlargement.^1^

Calcium Channel Blockers (CCB) have been widely used for the management of cardiovascular diseases including hypertension, angina pectoris and cardiac arrythmias and reported to be associated with gingival enlargement since 1984.^2,3^ Among calcium channel blockers, nifedipine is commonly associated with gingival enlargement with a reported prevalence of 14.7-83%,^4^ however, it is relatively less associated with amlodipine, as compared with nifedipine.^5^ In the context of Nepal, CCB (Particularly amlodipine) is the most frequently prescribed anti-hypertensive medication as a mono-therapy.^6^ Therefore, it is crucial, to patients benefit, that the clinicians must be aware of the adverse effects associated with such drug.

The present study aims to determine the prevalence of gingival enlargement in patients under amlodipine therapy in a tertiary care hospital and its association with drug dosage and the duration of drug intake by the patient.

## METHODS

This is a single-centered descriptive cross-sectional study, carried out in Dhulikhel Hospital. The participants were screened/diagnosed in the Department of Internal Medicine and sent to the Department of Periodontology and Oral Implantology for further investigations. The study period was from December, 2023 to May, 2024. Ethical clearance was obtained from the Institutional Review Committee (IRC) of Dhulikhel Hospital (Reference No. 242/23). Informed consent was taken from the patients prior to the study. The sample size was determined based on prevalence method using data derived from a similar study.^7^ Sample size was calculated by using 95% confidence level. Formula used was:


$$\begin{array}{l} \text{n}={\text{Z}}^{\text{2}}\times \frac{\text{p}\times \text{q}}{{\text{e}}^{\text{2}}}\\ \;=1.96^{2}\times \frac{\text{0.034 }\times \text{ 0.966}}{0.017^{2}}\\ \;=\frac{\text{3.84 }\times \text{ 0.032844}}{\text{0.000289}}\\ \;=436\sim 450\;(\text{adjusted for non-response}) \end{array}$$

Where,

n = sample size
Z = 1.96
p = Prevalence, taken as 3.4%
q = 1-p
e = standard error (Since p < 10%, e = p/2)

Patients above the age of 20 years, under Amlodipine therapy for at least six months, with presence of at least 16 permanent teeth and a minimum of 10 anterior teeth were included in the study. Edentulous patients, patients taking medications other than Amlodipine known to be associated with gingival enlargement as well as patients who had undergone periodontal treatment in the previous six months were excluded from the study. Also, patients with known conditions or diseases that can cause gingival enlargement such as pregnancy, leukemia and granulomatous disease were excluded. Patients who refuse to provide the informed consent were not included.

The examination was confined to the 12 most anterior teeth (Right canine to left canine in both maxilla and mandible). The demographic data including the age and gender of the patient were recorded and information regarding the drug such as drug dosage and duration of drug intake were also obtained from the patient. Gingival enlargement on each patient was assessed using the gingival enlargement scoring index by Bokenkamp and Bonhorst.^8^

Grade 0 → No signs of gingival enlargement
Grade 1 → Enlargement confined to interdental papilla
Grade 2 → Enlargement involving interdental papilla and marginal gingiva
Grade 3 → Enlargement covering three quarters or more of the crown

All the data were entered in Microsoft Excel version 2016 and analyzed using IBM SPSS Statistics for Windows, version 20 (IBM Corp., Armonk, N.Y., USA). Descriptive statistics were measured as mean and standard deviation.

## RESULTS

A total of 450 participants were included in this study, out of which 205 (45.60%) were male and 245 (54.40%) were female. The mean age group of the participants was 34.24±13.81 years. Among 450 participants under amlodipine therapy, the proportion of amlodipine-induced gingival enlargement was 129 (28.70%). Out of 205 male, amlodipine-induced gingival enlargement was prevalent in 58 (28.29%) and prevalence in female was in 71 (28.97%), out of 245. The prevalence of gingival enlargement, according to the grading, dosage, and duration of drug intake is given below. Grade 1 gingival enlargement was found to be most prevalent, 93 (20.70%) while grade 3 was seen in only 6 (0.90%) of the patients (Table 1). The severity of gingival enlargement was found to be increased with the increasing dosage (Table 2) and the duration of drug intake (Table 3).

**Table 1:** Prevalence of amlodipine-induced gingival enlargement according to the grading. (n=450)

Grading of gingival enlargement	Prevalence, n (%)
Grade 0	321 (71.30)
Grade 1	93 (20.70)
Grade 2	30 (7.10)
Grade 3	6 (0.90)

**Table 2:** Prevalence of amlodipine-induced gingival enlargement based on the drug dosage taken by the patient

Drug dosage	Study population	Prevalence, n(%)
2.5 mg	11	1 (9.09%)
5 mg	370	88 (23.70%)
7.5 mg	9	5 (55.50%)
10 mg	60	35 (58.33%)

**Table 3:** Prevalence of amlodipine-induced gingival enlargement according to duration of drug intake by the patient

Duration of drug intake	Study population	Prevalence, n(%)
<5 years	226	19 (8.40%)
years	136	50 (36.76%)
>10 years	88	60 (68.18%)

## DISCUSSION

The present study aimed to determine the prevalence of amlodipine-induced gingival enlargement in a tertiary care centre, examining its relationship with enlargement grade, drug dosage, and duration of intake. The prevalence of amlodipine-induced gingival enlargement was 129 patients (28.70%). Gender did not influence the prevalence, whereas higher dosages and longer duration of intake coincided with increased rates of enlargement. The most common dosage was 5 mg, taken by 370 of the 450 patients.

The CCBs are the frequently prescribed medications in the treatment of the wide range of cardiovascular diseases.^9^ Among these broad group of medications, dihydropyridines are commonly associated with the untoward effect of gingival enlargement.^10^ Amlodipine represents a class of 3^rd^ generation dihydropyridine which shares structural similarities with nifedipine, however, less association with gingival enlargement as compared to nifedipine.^5,11^ The first incidence of amlodipine-induced gingival enlargement was reported in 1994, in a report of three cases, who were under amlodipine therapy.^10^

The mechanism of action of CCBs, which induce gingival enlargement is poorly understood. It is thought to be involved in impeding the calcium ion influx through the smooth and cardiac muscle cell membranes, consequently preventing the intracellular mobilization of calcium.^12^ An anticonvulsant (phenytoin) and CCBs share a common mechanism of action, relating to their ability to influence calcium metabolism, despite their distinct pharmacological effects.^12^ Through disruption of calcium metabolism, they interfere with T-cell proliferation or activation by decreasing Ca++level, thereby upregulating gingival fibroblasts and increasing collagen biosynthesis. Another suggested mechanism for the gingival overgrowth could be over-synthesis of extracellular ground substance as evidenced by higher levels of collagen and sulphated mucopolysaccharides (glycosaminoglycans), as well as higher concentration of active fibroblasts.^13^ The increased collagen synthesis may also be attributed to the reduction in folic acid uptake by the gingival fibroblasts, causing alterations in the production of matrix metalloproteinases and the inability to activate collagenase, leading to gingival enlargement.^14^

The present study shows a 28.7% prevalence of gingival enlargement induced by Amlodipine, which is inconsistent with the findings by Jorgensen et al (3.3%) and Kothari et al (4.6%).^5,15^ This discrepancy could be due to the inclusion criteria of the study by Jorgensen including patients who had only taken amlodipine 5mg, whereas the current study includes patients taking any dose of amlodipine. In our study, among the patients who take Amlodipine 10 mg daily, 58% had gingival enlargement. There could be an association with the higher dose of amlodpine and the onset of gingival enlargement. This is also corroborated by another hospital based study where in the patients taking a lower dose of amlodpine 2.5 mg, gingival hyperplasia was absent.^15^ In our study, only one out of the eleven patients taking 2.5 mg amlodpine had ginginval hyperplasia.

The prevalence of gingival enlargement is also found to be significantly associated with the drug dosage and duration of drug intake by the patient, which is in accordance with the findings obtained from the similar study done by Rajkarnikar et al and others.^16-18^ Gingival enlargement does not occur in all patients receiving the same amount of medication. Possible explanation for this could be abnormal susceptibility of the gingival fibroblasts to the medication in some individuals.

The treatment option for drug induced gingival enlargement should be designed, keeping in mind, the medication being used and the clinical characteristics of the case. Consultation with the physician and possibility for drug discontinuation and its replacement should always be considered. Hence, treatment includes non-surgical periodontal therapy, drug substitution followed by surgical therapy (in severe cases).^19,20,21^ When trying to attempt a drug substitution, a time period of 6-12 months should be allowed between discontinuation of the drug and potential resolution of the enlargement.^19^

The limitations of the present study include the shorter time duration of the study and the samples collected from the limited population so the finding for the whole population may not be derived. It is not clear that whether the gingival enlargement is solely the result of the drug or other confounding factors such as the oral hygiene of the patient may have played a role. Taking this into consideration, further larger scale studies may be required including the patient's oral hygiene status as well as the histopathological examination to clearly understand the disease process.

## CONCLUSION

Within the limitations of the study, the present study showed that gingival enlargement could be the potential adverse effect associated with the usage of amlodipine. From this study, it is possible to state that the degree and severity of gingival enlargement has a correlation with the drug dosage and duration of drug intake by the patient.
